# Staple Foods Consumption and Irritable Bowel Syndrome in Japanese Adults: A Cross-Sectional Study

**DOI:** 10.1371/journal.pone.0119097

**Published:** 2015-03-18

**Authors:** Zhaoqiu Zheng, Cong Huang, Yinting Guo, Kaijun Niu, Haruki Momma, Yoritoshi Kobayashi, Shin Fukudo, Ryoichi Nagatomi

**Affiliations:** 1 Department of Medicine and Science in Sports and Exercise, Tohoku University Graduate School of Medicine, Sendai, Japan; 2 Division of Biomedical Engineering for Health and Welfare, Tohoku University Graduate School of Biomedical Engineering, Sendai, Japan; 3 Department of Behavioral Medicine, Tohoku University Graduate School of Medicine, Sendai, Japan; 4 Department of Epidemiology, School of Public Health, Tianjin Medical University, 22 Qixiangtai Road, Heping District, Tianjin, China; Geisel School of Medicine at Dartmouth College, UNITED STATES

## Abstract

**Background:**

Carbohydrates can cause gastrointestinal symptoms due to incomplete absorption in the small bowel. Thus, high-carbohydrate diets may induce symptoms of irritable bowel syndrome (IBS).

**Objective:**

This observational and cross-sectional study assessed the association between consumption of several carbohydrate-enriched staple foods, such as rice, Japanese wheat noodles, Chinese noodles, bread, pasta, and buckwheat noodles, and the prevalence of IBS in Japanese adults.

**Subjects and Methods:**

One thousand and eighty-two (837 men) Japanese adult employees aged 19-85 were included in this cross-sectional study conducted in 2011. IBS diagnosis was based on the Rome III criteria. Consumption of staple foods was assessed using a brief self-administered diet history questionnaire, and divided into three categories (low, middle, high) depending on their distribution.

**Results:**

In the multivariate analysis, daily consumption of rice (odds ratios [ORs] and [95% confidence interval (CI)]: middle, 1.36 [0.93–1.99]; high, 1.67 [1.12–2.49]; *P* for trend = 0.01), bread (middle, 1.88 [1.28–2.75]; high, 1.63 [1.10–2.41]; *P* for trend = 0.01), pasta (middle, 1.47 [1.01–2.15]; high, 1.68 [1.12–2.52]; *P* for trend = 0.01), and buckwheat noodles (middle, 1.76 [1.18–2.61]; high, 1.98 [1.31–3.00]; *P* for trend = 0.001) were associated with higher prevalence of IBS after adjustment for socio-demographic, anthropometric, and lifestyle-related factors. Buckwheat noodles, but not other staple foods, retained an association with the prevalence of IBS even after adjustment for daily intake of carbohydrates or plant proteins.

**Conclusions:**

This cross-sectional study demonstrated that the consumption of staple foods, such as rice, bread, pasta, and buckwheat noodles is associated with the prevalence of IBS. Of these, the consumption of buckwheat noodles, but not other staple foods, is associated with IBS independent of carbohydrate or plant protein contents.

## Introduction

Irritable bowel syndrome (IBS) is a common functional gastrointestinal disorder associated with abdominal pain or discomfort [1]. The prevalence of IBS in Japan was 13.1% among the general population [2], and 31% among outpatients [3]. In Western countries, the incidence of IBS is between 3% and 20% [4–6]. Observational studies showed that patients with IBS tended to have lower health-related quality of life [7, 8], suggesting the importance of preventing and controlling IBS to improve general health status.

The precise cause of IBS, however, is still unclear. High-carbohydrate diets have been shown to play a role in the symptoms of IBS, because carbohydrates could cause gastrointestinal symptoms due to incomplete absorption in the small bowel [9]. Indeed, previous studies have revealed that very low-carbohydrate diets can improve the diarrhea-predominant symptoms of IBS [10]. Similarly, Staudacher *et al*. [11, 12] reported that a low fermentable carbohydrate diet appeared to be more effective than standard dietary advice for symptom control of IBS.

Staple foods in Japan generally consist of rice, wheat, and buckwheat as the major source of carbohydrate; thus, it is possible that these staple foods are associated with the prevalence of IBS. Of these staple foods, it is worth noting that buckwheat is known as a high allergenic food because it contains several allergenic proteins [13, 14]. A nation-wide survey in Japan showed that 56 patients (33%) of 169 with buckwheat allergosis had gastrointestinal disorders, suggesting a potential involvement of buckwheat-induced allergic reaction in gut symptoms [15]. To date, however, very few studies have investigated the possible association between buckwheat or other staple foods and IBS prevalence.

This population-based cross-sectional study therefore aims to examine whether the consumption of major staple foods in Japan, including rice, Japanese wheat noodles, Chinese noodles, bread, pasta, and buckwheat noodles, are associated with the prevalence of IBS in Japanese adults.

## Methods

### Study population

This cross-sectional study is a part of the Oroshisho Study, a prospective study of lifestyle-related illnesses and health status in Japanese adult workers aged 19–85 years based on annual health examinations for employees between 2008 and 2011. In 2011, an additional assessment of IBS was conducted that requested information on diet, physical activity (PA), depressive symptoms, and other lifestyle-related factors in addition to the annual health examination. The details of this study are described elsewhere [16, 17]. The current study used data obtained in 2011 due to the availability of information on IBS prevalence.

In the 1 206 participants who underwent the health examinations, 1 163 provided their written informed consent to participate in this study. Of these, 81 subjects were excluded from analysis because their data on IBS (n = 47), dietary data (n = 21) or others (n = 13) were not available. Thus, 1 082 participants were included in the final analysis (837 men). All research procedures in the current study were consistent with ethical principles for medical research involving human subjects set by the Declaration of Helsinki [18]. The protocol for our study was approved by the institutional review board of the Tohoku University Graduate School of Medicine.

### Assessment of staple foods and other dietary content

Staple foods consumed during the past month before the examination, including rice, Japanese wheat noodles (udon), Chinese noodles (ramen), bread, pasta, and buckwheat noodles (soba) were estimated based on the results of a brief self-administered diet history questionnaire (BDHQ) containing questions about the frequency of consumption of 75 principal foods [19]. The consumption of energy and nutrients such as fat, plant protein, carbohydrate, soluble fiber, and insoluble fiber were also assessed by the BDHQ. The mean daily consumption of staple foods, energy, and nutrients was calculated using an *ad hoc* computer program developed to analyze the questionnaire. Consumption of staple foods was divided into three categories (low, middle, high) depending on their distribution. The reproducibility and validity of the BDHQ have been described in detail elsewhere [20, 21].

### Diagnosis of IBS

The diagnosis of IBS in this study was based on the Rome III criteria [1], in which IBS was diagnosed as follows: at least 3 days per month in the last 3 months of abdominal discomfort or pain that has 2 of 3 features: (1) improvement with defecation; and/or (2) onset associated with a change in frequency of stool; and/or (3) onset associated with a change in form of stool.

### Relevant covariates

Height and weight were measured using a standardized protocol. Body mass index (BMI) was calculated as weight (kg) divided by height squared (m^2^). The International Physical Activity Questionnaire was used to assess and calculate total PA [22]. PA was divided into two categories: <23 and ≥23 metabolic equivalent (MET) hours/week. More than 23 MET hours/week is the reference amount of PA for the prevention of lifestyle-related diseases, which is recommended by the Ministry of Health, Labour and Welfare of Japan based on a systematic review of epidemiological studies worldwide [23]. The Japanese version of the Self-Rating Depression Scale was used to examine the subjective severity of depression [24]. Participants who scored ≥45 raw sum points were regarded as having depression [25]. Information on age, sex, smoking status (never, former, current), drinking frequency (never, sometimes, every day), education levels (<college or ≥college), occupation (desk work or not), and sleep duration (6–8 hours/day or not) were gathered with a self-administered questionnaire survey.

### Statistical analysis

Data in this study were expressed as means (standard deviation [SD] or 95% confidence interval [CI]) or odds ratios (ORs; 95% CI) for continuous variables and percentages for categorical variables. In this study, all continuous variables were log-transformed prior to multivariate statistical analyses due to their abnormal distribution and back-transformed for data presentation. The variables neared normal distribution after log-transformation. Comparison of participant characteristics between subjects with IBS and those without IBS was performed through an analysis of covariance (ANCOVA) or a logistic regression analysis. Difference in consumption of each staple food between the IBS group and non-IBS group was examined with an ANCOVA. Multivariate associations between staple foods and prevalence of IBS were examined by logistic regression analysis. In this multivariate analysis, Model 1 was adjusted for age, sex, and BMI. Model 2 was additionally adjusted for smoking status, drinking frequency, occupation, educational levels, sleep duration, PA, and depressive symptoms. Other models were additionally adjusted for daily intake of fat (Model 3), carbohydrate (Model 4), plant protein (Model 5), soluble fiber (Model 6), or insoluble fiber (Model 7). The ORs and 95% CI of IBS compared with the lowest level of staple food consumption as the reference was calculated to examine the *P* value for linear trend. All statistical tests were two-tailed and *P* values < 0.05 were considered statistically significant. Data were analyzed with IBM SPSS Statistics 19.0 software (IBM SPSS Inc., Chicago, IL, USA).

## Results

### Participant characteristics

In this study, the mean age (SD) of participants was 46.1 (11.2) years (Table 1). The prevalence of IBS was 19.6% (212 of 1 082), with 149 men (17.8%) and 63 women (25.7%).

**Table 1:** Participant characteristics.

Number of participants	1 082
Age (years) ^a^	46.1 ± 11.2
Male (%)	77.4
BMI (kg/m^2^)	23.4 ± 3.7
Staple foods consumption	
Rice (g/day)	306 ± 176
Japanese wheat noodles (g/day)	28.8 ± 26.1
Chinese noodles (g/day)	31.7 ± 33.6
Bread (g/day)	30.9 ± 26.6
Pasta (g/day)	17.4 ± 18.2
Buckwheat noodles (g/day)	28.3 ± 27.5
Energy and nutrient intakes	
Energy (kcal/day)	1875 ± 622
Fat (g/day)	48.6 ± 19.7
Carbohydrate (g/day)	249 ± 92
Plant protein (g/day)	29.0 ± 10.3
Soluble fiber (g/day)	2.69 ± 1.33
Insoluble fiber (g/day)	7.8 ± 3.5
Smoking status	
Former (%)	13.5
Current (%)	39.9
Drinking frequency	
Sometimes (%)	48.9
Every day (%)	27.7
Education levels ≥college (%)	32.8
Desk work (%)	63.2
Sleep duration 6–8 hours/day (%)	78.1
Depressive symptoms (%)	33.2
PA ≥23 MET hours/week (%)	34.3
IBS diagnosis (%)	19.6

BMI, body mass index; IBS, irritable bowel syndrome; PA, physical activity; MET, metabolic equivalent.

^a^ Variables were presented as means ± standard deviation or percentages.

Table 2 shows the age- and sex-adjusted differences in participant characteristics between the IBS diagnosed and non-IBS groups. The mean age of the IBS group was significantly lower than that of the non-IBS group after adjusting for sex (*P* < 0.05). Women were more likely to have IBS compared with men (*P* < 0.05). Additionally, depressive symptoms were associated with IBS prevalence (*P* < 0.01). For daily energy and nutrient intakes, energy, carbohydrate, and plant protein were positively associated with the prevalence of IBS (*P* ≤ 0.01). Furthermore, subjects with IBS had a higher consumption of rice, bread, pasta, and buckwheat noodles than those without IBS (*P* < 0.05). No other differences between the two groups were observed.

**Table 2:** Age- and sex- adjusted participant characteristics between IBS group and non-IBS group.^a^

	IBS group	Non-IBS group	*P* value
	(n = 212)	(n = 870)	
Age (years) ^b^ ^,^ ^c^	42.6 (41.2–44.1)	45.2 (44.5–46.0)	0.002
Male (%)	70.3	79.1	0.016
BMI (kg/m^2^) ^c^	22.9 (22.4–23.3)	23.1 (22.9–23.4)	0.329
Staple foods consumption ^c^			
Rice (g/day)	288 (258–321)	241 (228–254)	0.004
Japanese wheat noodles (g/day)	18.6 (15.8–21.9)	17.8 (16.4–19.3)	0.629
Chinese noodles (g/day)	17.8 (14.7–21.5)	15.7 (14.3–17.2)	0.249
Bread (g/day)	22.5 (19.1–26.4)	18.1 (16.7–19.6)	0.019
Pasta (g/day)	11.5 (9.5–13.8)	8.9 (8.1–9.8)	0.020
Buckwheat noodles (g/day)	19.3 (16.2–23.0)	15.1 (13.8–16.5)	0.015
Energy and nutrient intakes ^c^			
Energy (kcal/day)	1861 (1782–1946)	1751 (1713–1789)	0.014
Fat (g/day)	46.7 (44.2–49.4)	44.3 (43.1–45.6)	0.102
Carbohydrate (g/day)	249 (237–262)	228 (222–233)	0.001
Plant protein (g/day)	28.9 (27.5–30.3)	26.8 (26.2–27.4)	0.006
Soluble fiber (g/day)	2.49 (2.32–2.66)	2.36 (2.28–2.44)	0.188
Insoluble fiber (g/day)	7.0 (6.8–7.2)	7.4 (7.0–7.9)	0.103
Smoking status			
Former (%)	17.5	12.5	0.087
Current (%)	35.8	40.9	0.784
Drinking frequency			
Sometimes (%)	49.5	48.7	0.706
Every day (%)	25.0	28.4	0.918
Education levels ≥college (%)	27.8	34.0	0.091
Desk work (%)	63.7	63.1	0.729
Sleep duration 6–8 hours/day (%)	75.0	78.9	0.322
Depressive symptoms (%)	42.5	30.9	0.002
PA ≥23 MET hours/week (%)	32.5	34.7	0.908

BMI, body mass index; IBS, irritable bowel syndrome; PA, physical activity; MET, metabolic equivalent.

^a^ Analyzed by analysis of covariance or logistic regression analysis.

^b^ Variables were presented as means (95% confidence interval) or percentages.

^c^ Continuous variables were log-transformed for analysis and back-transformed for data presentation.

### Association between staple foods and IBS prevalence

**Fig 1:**
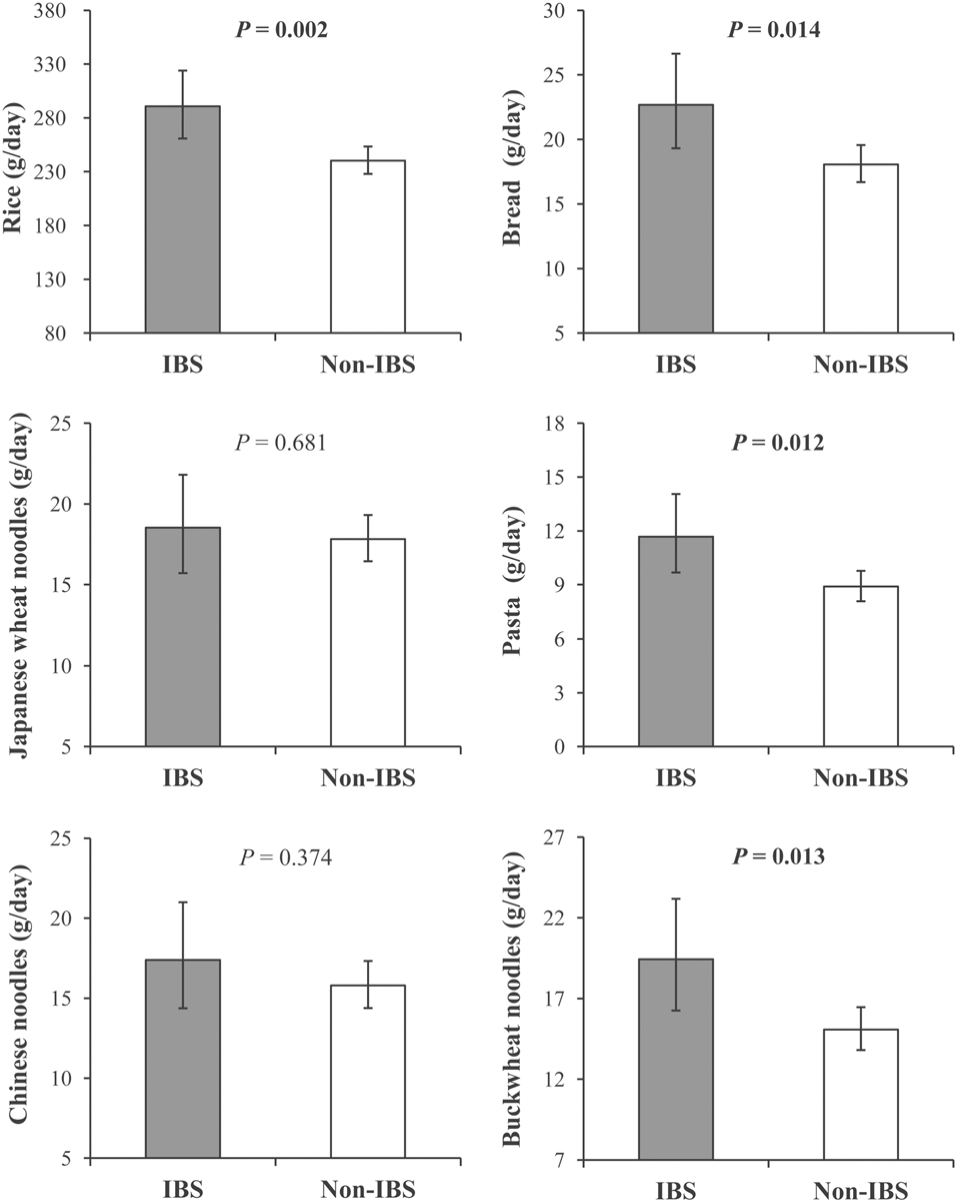
Analysis of Covariance on Association between Staple Foods Consumption and Irritable Bowel Syndrome (IBS) Prevalence. Confounding factors include age (continuous variable), sex, body mass index (continuous variable), smoking status (never, former, current), drinking frequency (never, sometimes, every day), occupation (desk work), educational levels (≥college), sleep duration (6–8 hours/day), physical activity (≥23 metabolic equivalent hours/week), and depressive symptoms (self-rating depression scale ≥45 points). Data on the consumption of staple foods were log-transformed prior to multivariate statistical analyses due to their abnormal distribution and back-transformed for data presentation. Data were shown as means and 95% confidence interval. Of the 1 082 subjects, 212 had IBS.

ANCOVA showed that the consumption of rice, bread, pasta, and buckwheat noodles were positively associated with the prevalence of IBS after adjusting for age, sex, BMI, smoking status, drinking frequency, occupation, educational levels, sleep duration, PA, and depressive symptoms (*P* < 0.05) (Fig. 1).

In logistic regression analysis, as shown in Table 3, the ORs [95% CI] of IBS prevalence across these staple foods in Model 2 were as follows: rice (low, 1.00 [reference]; middle, 1.36 [0.93–1.99]; high, 1.67 [1.12–2.49]; *P* for trend = 0.012), bread (middle, 1.88 [1.28–2.75]; high, 1.63 [1.10–2.41]; *P* for trend = 0.014), pasta (middle, 1.47 [1.01–2.15]; high, 1.68 [1.12–2.52]; *P* for trend = 0.010), and buckwheat noodles (middle, 1.76 [1.18–2.61]; high, 1.98 [1.31–3.00]; *P* for trend = 0.001), respectively. Similarly, IBS prevalence was also higher across the tertiles of Chinese noodles, although this association was non-significant (*P* = 0.069). No association between Japanese wheat noodles and IBS was found. Further, the association between buckwheat noodles consumption and the prevalence of IBS remained significant even after additional adjustment for daily intakes of fat, carbohydrate, plant protein, soluble fiber, or insoluble fiber (*P* for trend < 0.05 for all models). In contrast, no significant association between the consumption of rice, bread, and pasta, and the prevalence of IBS was observed when the model was adjusted for carbohydrate or plant protein intake.

**Table 3:** Odds ratios (95% confidence interval) of IBS prevalence in staple foods consumption.^a^

	Consumption of staple foods			*P* for trend
	Low	Middle	High	
Rice (g/day)	<208	208–351	>351	
Participants (n)	367	373	342	
Model 1	1.00 (Reference)	1.30 (0.89–1.89)	1.61 (1.09–2.39)	0.017
Model 2	1.00 (Reference)	1.36 (0.93–1.99)	1.67 (1.12–2.49)	0.012
Model 3	1.00 (Reference)	1.33 (0.91–1.59)	1.59 (1.05–2.39)	0.026
Model 4	1.00 (Reference)	1.14 (0.76–1.72)	1.09 (0.64–1.87)	0.722
Model 5	1.00 (Reference)	1.25 (0.85–1.85)	1.33 (0.85–2.01)	0.200
Model 6	1.00 (Reference)	1.34 (0.91–1.96)	1.60 (1.07–2.40)	0.023
Model 7	1.00 (Reference)	1.31 (0.90–1.93)	1.54 (1.01–2.33)	0.043
Japanese wheat noodles (g/day)	<12.93	12.93–25.41	>25.41	
Participants (n)	369	393	320	
Model 1	1.00 (Reference)	1.31 (0.91–1.89)	1.12 (0.76–1.66)	0.523
Model 2	1.00 (Reference)	1.32 (0.91–1.92)	1.11 (0.75–1.64)	0.585
Model 3	1.00 (Reference)	1.29 (0.89–1.86)	1.03 (0.69–1.54)	0.856
Model 4	1.00 (Reference)	1.30 (0.90–1.89)	0.94 (0.63–1.42)	0.839
Model 5	1.00 (Reference)	1.27 (0.88–1.84)	0.92 (0.61–1.39)	0.742
Model 6	1.00 (Reference)	1.28 (0.88–1.86)	1.01 (0.67–1.52)	0.914
Model 7	1.00 (Reference)	1.27 (0.88–1.85)	0.99 (0.66–1.48)	0.989
Chinese noodles (g/day)	<10.78	10.78–25.41	>25.41	
Participants (n)	387	339	356	
Model 1	1.00 (Reference)	1.31 (0.89–1.93)	1.51 (1.03–2.22)	0.037
Model 2	1.00 (Reference)	1.24 (0.84–1.84)	1.44 (0.97–2.14)	0.069
Model 3	1.00 (Reference)	1.21 (0.82–1.80)	1.36 (0.91–2.04)	0.079
Model 4	1.00 (Reference)	1.15 (0.77–1.71)	1.23 (0.82–1.85)	0.330
Model 5	1.00 (Reference)	1.18 (0.79–1.75)	1.23 (0.81–1.86)	0.334
Model 6	1.00 (Reference)	1.23 (0.83–1.82)	1.36 (0.91–2.04)	0.133
Model 7	1.00 (Reference)	1.22 (0.83–1.81)	1.35 (0.90–2.01)	0.144
Bread (g/day)	<11.55	11.55–31.76	>31.76	
Participants (n)	407	335	340	
Model 1	1.00 (Reference)	1.83 (1.26–2.67)	1.60 (1.09–2.24)	0.017
Model 2	1.00 (Reference)	1.88 (1.28–2.75)	1.63 (1.10–2.41)	0.014
Model 3	1.00 (Reference)	1.81 (1.23–2.68)	1.54 (1.02–2.32)	0.042
Model 4	1.00 (Reference)	1.70 (1.15–2.51)	1.38 (0.91–2.09)	0.134
Model 5	1.00 (Reference)	1.73 (1.17–2.55)	1.40 (0.92–2.11)	0.121
Model 6	1.00 (Reference)	1.83 (1.25–2.69)	1.56 (1.04–2.32)	0.031
Model 7	1.00 (Reference)	1.81 (1.23–2.66)	1.53 (1.02–2.29)	0.038
Pasta (g/day)	<9.70	9.70–20.79	>20.79	
Participants (n)	403	371	308	
Model 1	1.00 (Reference)	1.40 (0.96–2.03)	1.63 (1.10–2.43)	0.015
Model 2	1.00 (Reference)	1.47 (1.01–2.15)	1.68 (1.12–2.52)	0.010
Model 3	1.00 (Reference)	1.43 (0.97–2.09)	1.58 (1.04–2.41)	0.030
Model 4	1.00 (Reference)	1.37 (0.93–2.01)	1.45 (0.96–2.20)	0.077
Model 5	1.00 (Reference)	1.38 (0.94–2.02)	1.44 (0.94–2.20)	0.086
Model 6	1.00 (Reference)	1.44 (0.99–2.11)	1.59 (1.05–2.42)	0.025
Model 7	1.00 (Reference)	1.43 (0.98–2.09)	1.56 (1.03–2.37)	0.033
Buckwheat noodles (g/day)	<10.78	10.78–25.41	>25.41	
Participants (n)	363	401	318	
Model 1	1.00 (Reference)	1.78 (1.21–2.63)	2.00 (1.32–3.01)	0.001
Model 2	1.00 (Reference)	1.76 (1.18–2.61)	1.98 (1.31–3.00)	0.001
Model 3	1.00 (Reference)	1.72 (1.15–2.56)	1.89 (1.24–2.89)	0.004
Model 4	1.00 (Reference)	1.70 (1.14–2.53)	1.76 (1.15–2.68)	0.010
Model 5	1.00 (Reference)	1.69 (1.13–2.51)	1.72 (1.11–2.65)	0.015
Model 6	1.00 (Reference)	1.74 (1.17–2.58)	1.88 (1.23–2.89)	0.004
Model 7	1.00 (Reference)	1.72 (1.16–2.56)	1.84 (1.20–2.83)	0.006

*Notes*: Model 1, adjusted for age (continuous variable), sex, and body mass index (continuous variable); Model 2, adjusted for Model 1 + smoking status (never, former, current), drinking frequency (never, sometimes, every day), occupation (desk work), educational levels (≥college), sleep duration (6–8 hours/day), physical activity (≥23 metabolic equivalent hours/week), and depressive symptoms (self-rating depression scale ≥45 points); Model 3, adjusted for Model 2 + fat intake (continuous variable); Model 4, adjusted for Model 2 + carbohydrate intake (continuous variable); Model 5, adjusted for Model 2 + plant protein intake (continuous variable); Model 6, adjusted for Model 2 + soluble fiber intake (continuous variable); Model 7, adjusted for Model 2 + insoluble fiber intake (continuous variable).

^a^ Analyzed by logistic regression analysis.

## Discussion

This population-based cross-sectional study found a positive association between the prevalence of IBS and the consumption of rice, bread, pasta, and buckwheat noodles, even after adjusting for socio-demographic, anthropometric, and lifestyle-related factors, among Japanese adults. Interestingly, although the association between most staple foods and the prevalence of IBS could be attributed to total carbohydrate and plant protein intake, buckwheat noodles consumption was associated with the prevalence of IBS independent of these nutrients.

The finding of an association between the consumption of rice and wheat (Chinese noodles, bread, and pasta) and the prevalence of IBS in our study supports several previous reports. Rice and wheat are considered high carbohydrate foods, which have previously been linked to gastrointestinal symptoms in IBS. In a previous study with 330 IBS patients and 80 healthy volunteers, symptoms of bloating, abdominal pain, and diarrhea have been shown to be associated with the intake of foods rich in carbohydrates [26]. Furthermore, Austin *et al*. [10] demonstrated that being on a very low-carbohydrate diet (20 g carbohydrate/day) for 4 weeks improved the symptoms of abdominal pain and stool habit among 13 patients with moderate to severe diarrhea-predominant IBS. These studies provide an explanation for our findings of a disappearance of the association between rice, wheat, and the prevalence of IBS after adjusting for daily carbohydrate intake. Additionally, an association between rice and wheat consumption, and the prevalence of IBS could also be mediated through plant proteins, since a confounding effect of plant proteins was observed in this study. First, although the specific plant protein that plays a role in this association cannot be specified in this study, rice has been considered as a common cause of food protein-induced enterocolitis syndrome in children, probably due to an allergy to rice [27]. Further, gluten, an allergenic plant protein contained in wheat, may be a candidate as well. Recently, a double-blind, randomized, placebo-controlled study indicated that patients with IBS without celiac disease achieved satisfactory symptom control with a gluten-free diet, suggesting the involvement of gluten in the gut symptoms [28]. These findings suggest that a positive association of rice and wheat with the prevalence of IBS may be due to their rich content of carbohydrate and allergenic plant proteins.

Bread, Chinese noodles, and Japanese wheat noodles are common wheat–contained foods in Japan. However, Japanese wheat noodles did not show any association with prevalence of IBS, which may due to the different protein content in wheat flour compared with that used in bread and Chinese noodles. Bread and Chinese-type noodles are generally made from strong wheat flour (bread flour), in which protein content comprises 10.5–12.5% of the total nutrients [29]. By contrast, medium wheat flour (protein content ranging from 8.0 to 9.5%) is used for Japanese wheat noodles [29], which may result in a low level of gluten. Therefore, the relatively lower allergenic protein content of Japanese wheat noodles may explain why it was not associated with the prevalence of IBS.

To the best of our knowledge, these results demonstrate, for the first time, that buckwheat noodles consumption is associated with the prevalence of IBS independent of a number of potential confounders, which suggests that the contribution of buckwheat to IBS symptoms is different from that of rice and wheat; thus, other possible mechanisms should be described to explain the observed results. Buckwheat is not taxonomically related to wheat or other cereals. Buckwheat contains several well-recognized allergenic proteins including buckwheat 16- and 24-kDa protein (BW16KD and BW24KD) [13, 14], suggesting a strong allergenic potential. Indeed, Nakamura and Yamaguchi [15] reported that one third of the studied 169 Japanese patients who suffered from buckwheat allergy had gastrointestinal symptoms. Another report revealed that IBS patients had a higher positive food skin prick test when exposed to 18 fresh foods, including buckwheat, compared to controls [30]. Therefore, it is logical to hypothesize that buckwheat noodles might be associated with IBS prevalence via an allergic mechanism. Although daily plant protein intake was controlled in our multivariate analysis, the overall plant protein intake used in this study may not be representative of buckwheat allergenic proteins because allergic proteins may elicit allergic reactions after consumption of very small quantities. It must be noted that those who reported any consumption of buckwheat noodles are unlikely to suffer from an obvious buckwheat allergy, which often requires medical intervention, because those with an allergy would avoid eating buckwheat noodles [13, 14].

In a regular Japanese diet, rice contributes to more than 50% of the total carbohydrate intake [31]. Therefore, the reason for the disappearance of a positive association of rice with IBS prevalence after adjustment for carbohydrate intake may be partly due to the major contribution of rice to total carbohydrate intake. Buckwheat noodles, on the other hand, contribute only between 5 and 10% of the total carbohydrate intake in a regular Japanese diet—a similar proportion to bread, pasta, Chinese noodles, and Japanese wheat noodles. Consequently, one of the arguments against the positive association of buckwheat noodles with IBS prevalence (independent of carbohydrate intake) is that this is due merely to the smaller contribution of buckwheat noodles to the total dietary carbohydrate intake. However, since the associations between IBS symptoms and the consumption of other staple foods with contributions to the total carbohydrate intake similar to that of buckwheat (such as Chinese noodles and bread) were dependent on carbohydrate, we consider the association of buckwheat noodles with IBS to be independent of total carbohydrate intake.

It is also worth mentioning that smoking habits did not show any association with IBS prevalence in this study, although the negative role of current smoking in IBS prevalence has been reported by other population-based studies [32, 33]. Meanwhile, association between staple foods and IBS prevalence was not affected by adjustment for smoking status in multivariate analysis, suggesting the observed association between several staple foods and IBS prevalence in this study is not mediated by smoking habits.

Two main limitations of this study should be noted. The first is the cross-sectional design of this study, which does not allow us to deduce a causal relationship from our findings. Thus, future prospective studies are needed to determine the association between the consumption of staple foods, especially buckwheat, and the incidence or exacerbation of IBS. Another limitation was that our study did not include details of allergenic proteins, such as gluten, BW16KD, and BW24KD in staple foods. These data may have enabled further interpretation of the findings observed.

In conclusion, this cross-sectional observational study has demonstrated that the consumption of major staple foods in Japan, such as rice, bread, pasta, and buckwheat noodles are associated with the prevalence of IBS in Japanese adults. Daily intakes of carbohydrate or overall plant protein may mediate the association of IBS prevalence with rice and wheat. Intake of buckwheat noodles was associated with IBS prevalence independent of carbohydrate and plant protein intake. Further studies are needed to confirm these observations and elucidate the mechanisms of these associations.
